# Trimetazidine inhibits liver fibrosis and hepatic stellate cell proliferation and blocks transforming growth factor-β (TGFβ)/Smad signaling *in vitro* and *in vivo*

**DOI:** 10.1080/21655979.2022.2047403

**Published:** 2022-03-07

**Authors:** Wenwen Ding, Danhua Zhou, Shimeng Zhang, Jiaping Qian, Lingxia Yang, Lei Tang

**Affiliations:** a Department of Gastroenterology, Suzhou Ninth People’s Hospital, Suzhou, Jiangsu, P.R. China; b Department of Gastroenterology, Zhangjiagang First People’s Hospital, Zhangjiagang, Jiangsu, P.R. China

**Keywords:** Hepatic stellate cells, liver fibrosis, trimetazidine, TGF-β/Smad

## Abstract

Trimetazidine (TMZ) has been used extensively to treat coronary artery disease and to reduce fibrosis. Liver fibrosis is a reversible process. However, the impacts of TMZ on liver fibrosis triggered by CCl_4_ and on hepatic stellate cells in liver fibrosis remain to be elaborated. In the current study, the liver fibrosis models were constructed by using CCl_4_-induced mice and TGF-β-induced hepatic stellate cells. The involvement of TMZ in liver fibrosis was subsequently investigated. In the CCl_4_-induced hepatic fibrosis mouse model, it was shown that the expression levels of alanine aminotransferase and aspartate aminotransferase were reduced after TMZ treatment; the expression levels of the extracellular matrix proteins colla1 and α-SMA were down-regulated; furthermore, the expression levels of TGFβ/Smad signaling proteins were inhibited. In TGF-β-induced hepatic stellate cells, compared to the TGF-β-induced group, cell proliferation and migration were inhibited after TMZ treatment; meanwhile, extracellular matrix protein and TGFβ/Smad signaling protein expression levels followed the same trend as in the hepatic fibrosis model. In conclusion, TMZ could block the TGFβ/Smad signaling in liver fibrosis model, with inhibiting liver fibrosis and hepatic stellate cell proliferation. This may broaden the application sphere of TMZ in liver fibrosis therapy.

## Graphical abstract

**Figure:**
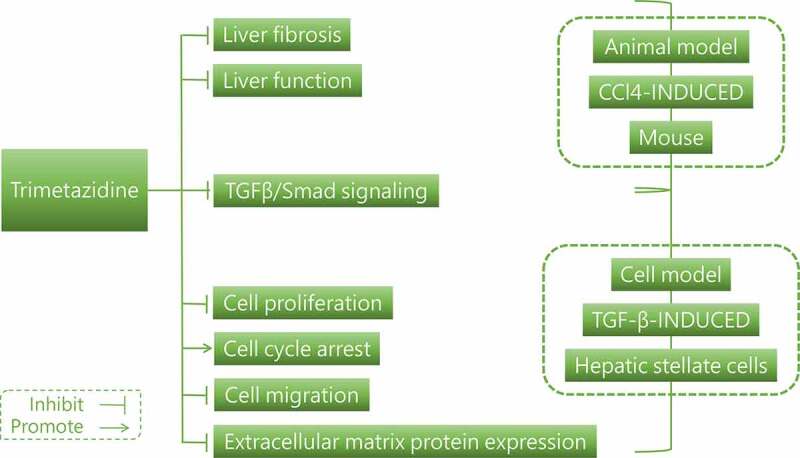

## Introduction

Chronic liver disease often occurs as in response to chronic injury and the inflammatory response in the liver, leading to liver fibrosis, cirrhosis and liver cancer [1–3]. Liver fibrosis arises from the redundant deposition of extracellular matrix proteins and is induced by two general types of chronic liver injury, including hepatotoxic injury resulting from chronic damage of hepatocytes and cholestatic injury resulting from impaired bile flow [3,4]. Of concern is that liver fibrosis is a reversible process [5]. Unless it has a tendency to lead to cirrhosis, the removal of the substances causing the fibrotic response will help the fibrosis to subside [1,6].

Trimetazidine (TMZ), a piperazine derivatives, has been widely used in the treatment of coronary artery disease, affecting myocardial substrate utilization by inhibiting oxidative phosphorylation and shifting energy metabolism from free fatty acid oxidation to glucose oxidation [7–9]. TMZ not only contributed to the reduction of free radical-induced injury, inhibition of apoptosis and improvement of endothelial function in myocardial disease but also had applications in coronary heart pathologies [10–12]. Studies have been reported that TMZ could reduce oxidative stress in the brain and liver of mice induced by lipopolysaccharide administration and be beneficial in the treatment of systemic inflammation [13,14]. In addition, TMZ has been demonstrated to improve ischemia-reperfusion-induced liver injury and to improve cisplatin-induced hepatotoxicity in rats [15–17]. However, whether TMZ could regulate CCl_4_-induced liver fibrosis still needs to be clarified. Meaningfully, hepatic stellate cells have been extensively reported as important target cells for liver fibrosis [18,19]. In earlier studies, the hepatic stellate cells were a chief source of myofibroblasts in liver parenchymal disease [20]. In addition, the TGFβ/Smad pathway, as a representative pathway involved in fibrogenesis, is often noticed in liver fibrosis [21].

In the present study, animal and cellular models of liver fibrosis were, respectively, constructed by CCl_4_-induced mice and TGF-β-induced hepatic stellate cells. Then the functions of TMZ on liver fibrosis were explored, and the effect of TMZ on TGFβ/Smad signaling pathway was studied *in vitro* and *in vivo*.

## Materials and methods

### Animal study

Fifteen C57BL/6 J male mice (6–8 weeks) were provided by Charles River Lab (Beijing, China). Before the start of the experiment, the animals were placed in an air-conditioned room at 25°C with a 12-hour light cycle. All animals were given proper care during the study. The experiment was approved by the Suzhou Ninth People’s Hospital Ethics Committee (Approval No. KY2021-049-01) and followed the ethical guidelines on the care and use of laboratory animals for this research.

Liver fibrosis model in mice was constructed through twice-weekly intraperitoneal injections of CCl_4_ (25%, 5 ml/kg) for 8 weeks. Mice were randomly assigned into three groups: Control group, CCl_4_ group and CCl_4_+ TMZ group (40 mg/kg, by gavage). After euthanasia of the mice, the livers were stripped, and half in liquid nitrogen were frozen rapidly and kept at −80°C. The other half were fixed in 4% paraformaldehyde sections for 24 h and paraffin embedded. Levels of liver enzyme including alanine aminotransferase (ALT; ab282882) and aspartate aminotransferase (AST; ab263882) in plasma samples were determined using corresponding ELISA kits from Abcam (Shanghai, China) in the light of the manufacturer’s guidance. For a more visual analysis of liver fibrosis, tissue sections were analyzed using Masson’s Trichrome Stain kit (Beijing Solarbio Technology Co., Ltd. China) according to the experiment manual.

### Western blotting

The treated tissues or cells were processed using lysis buffer, total protein was extracted and protein concentration was determined by the bicinchoninic acid method. The proteins were loaded onto polyvinylidene fluoride (PVDF) membranes following protein separation using 10% sodium dodecyl sulfate-polyacrylamide gel electrophoresis (SDS-PAGE). After the membranes were closed with 5% skimmed milk for 1 h, they were cultivated with primary antibody overnight at 4°C and subjected to secondary antibody hybridization at room temperature for 2 h the following day. Finally, signals were displayed with the aid of enhanced chemiluminescence (Thermo Fisher Scientific, Inc.). Image Lab Software (Bio-Rad, CA, USA) was adopted to determine the expression levels of proteins.

### Quantitative real-time PCR (RT-qPCR)

TRIzol reagent (Thermo Fisher Scientific, Inc.) was applied for the separation of total RNA from liver tissue or cells. cDNA was generated by using a Reverse Transcription Kit (Thermo Fisher Scientific, Inc.). And the iTaq Universal SYBR Green kit (Bio-Rad Laboratories, Inc. CA, USA) was then used to perform RT-qPCR. The study was performed with GAPDH as the internal reference gene. The 2^−ΔΔCq^ method was for relative quantification. The primer sequences used were as follows: colla1, mouse forward 5’-GGGGCAAGACAGTCATCGAA −3’ and reverse 5’-GAAGTAGACGGGGTTGAGGG −3’, human forward 5’-TGACGAGACCAAGAACTGCC-3’ and reverse 5’-GCACCATCATTTCCACGAGC −3’; α-SMA, mouse forward 5’-TGACTGAGCGTGGCTATTCC-3’ and reverse 5’-GCCAGGGCTACAAGTTAAGG −3’, human forward 5’-CCGGGACTAAGACGGGAATC-3’ and reverse 5’-ATGGGGACATTGTGGGTGAC −3’; GAPDH, mouse forward 5’-AGGTCGGTGTGAACGGATTTG-3’ and reverse 5’-GGGGTCGTTGATGGCAACA −3’, human forward 5’-CTGGGCTACACTGAGCACC-3’ and reverse 5’-AAGTGGTCGTTGAGGGCAATG −3’.

### Immunofluorescence analysis

Frozen sections of liver tissue were permeabilized with 0.5% TritonX-100 at room temperature for 15 min, followed by sealing the sections with 5% normal goat serum for 1 h at 37°C. Afterward, sections were hatched with the antibody targeting α-SMA (1:500) overnight at 4°C, and then immunostained with Alexa Fluor 488-conjugated secondary antibody. Finally, the sections were stained with DAPI at room temperature for 10 min and imaged by a fluorescent microscope (magnification ×200; Olympus, Beijing, China).

### Treatment of cells

Murine and human hepatic stellate cell lines (JS-1 and LX-2) were provided by BeNa Culture Collection (Henan, China) and cultured in Dulbecco’s modified Eagle medium (DMEM) supplemented with 1% penicillin/streptomycin and 10% FBS. All cells were maintained in a 37°C humidified atmosphere containing 5% CO_2_. For the TGF-β induction group (TGF-β), cells were insulted by 10 ng/ml TGF-β at 37°C for 48 h, followed by incubation with 10 μM or 40 μM TMZ for 30 min at 37°C, respectively, considered as the TMZ low (TGF-β+ TMZ low) and TMZ high (TGF-β+ TMZ high) groups [11,22]. Cells were digested by trypsin from the culture dish and diluted in phosphate buffer (PBS), and cell counts were determined using a hemocytometer.

### Cell cycle

JS-1 and LX-2 cells were inoculated in 6-well plates. Following cell apposition, cells were treated with TGF-β and/or TMZ. Subsequently, cells resuspended in 70% ethanol were fixed for 2 h after PBS washing. According to the cell cycle assay kit (Beyotime Biotechnology Inc. Shanghai, China) manual, cells were stained with staining solution containing PI and RNase A for 20 min at 4°C. And then, cell cycle distribution was detected by flow cytometry and analyzed with FlowJo V10.8 software (Swiss).

### Cell migration

Transwell migration assay was performed using 8 μm-well transwell plates. The upper chamber was inoculated with JS-1 and LX-2 cells, respectively, and in a serum-free medium. The bottom chamber contained medium in which 5% fetal bovine serum was added to promote cell migration. These chambers were incubated in an incubator at 37°C for 6 h. Subsequently the cells were fixed using 3.7% paraformaldehyde and stained with 0.1% crystalline violet. Using an inverted light microscope (magnification ×200), randomly selected areas were observed and photographed, and the number of invading cells was counted.

### Statistical analysis

One-way ANOVA and Tukey’s test were performed on the experimental data by using GraphPad Prism 8.0 software (GraphPad software, Inc.). Data were considered to be statistically different from each other when the *P* value was less than 0.05.

## Results

### TMZ inhibits the expression levels of extracellular matrix proteins and TGFβ/Smad signaling in mice liver fibrosis model imposed by CCl_4_

A mouse model of liver fibrosis was established utilizing CCl_4_ induction. Subsequently, mice were treated by TMZ and several series of tissue and protein-related assays were performed after euthanasia.

**Figure 1.**
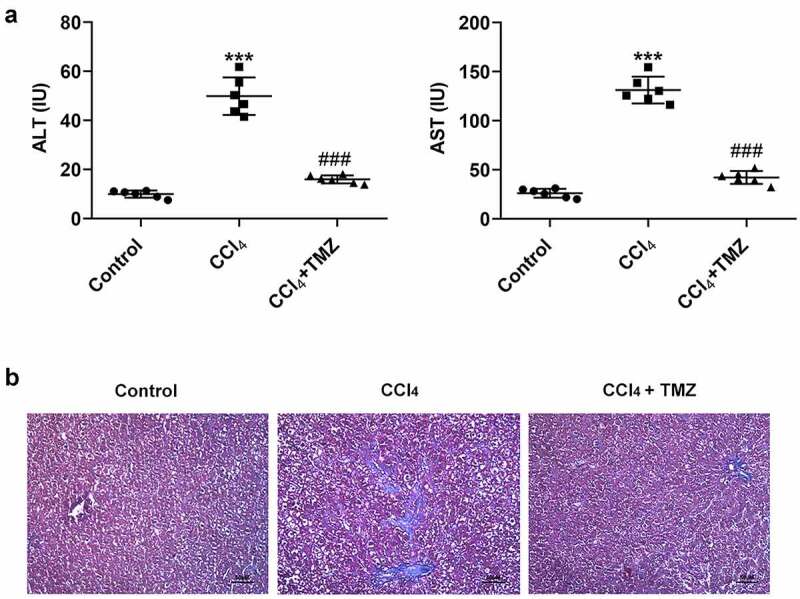
TMZ inhibits liver function changes and liver fibrosis in CCl_4_-induced mice. (a) Aminotransferase (ALT) and aminotransferase (AST) levels were measured by the kits. (b) Masson staining of tissue liver fibrosis. ***P < 0.001 vs. control; ^###^*P* < 0.001 vs. CCl_4._

The results exposed that the levels of liver enzymes (ALT and AST) were considerably increased in mice after CCl_4_ induction relative to the control group (Figure 1a). The marked feature of liver fibrosis was also found in the CCl_4_ group in the masson staining graph of liver tissue (Figure 1b). However, compared with the CCl_4_-induced group, after stimulated by TMZ, the ALT and AST expressions were significantly decreased and the fibrotic features of liver tissue were attenuated (Figure 1a and b).

**Figure 2.**
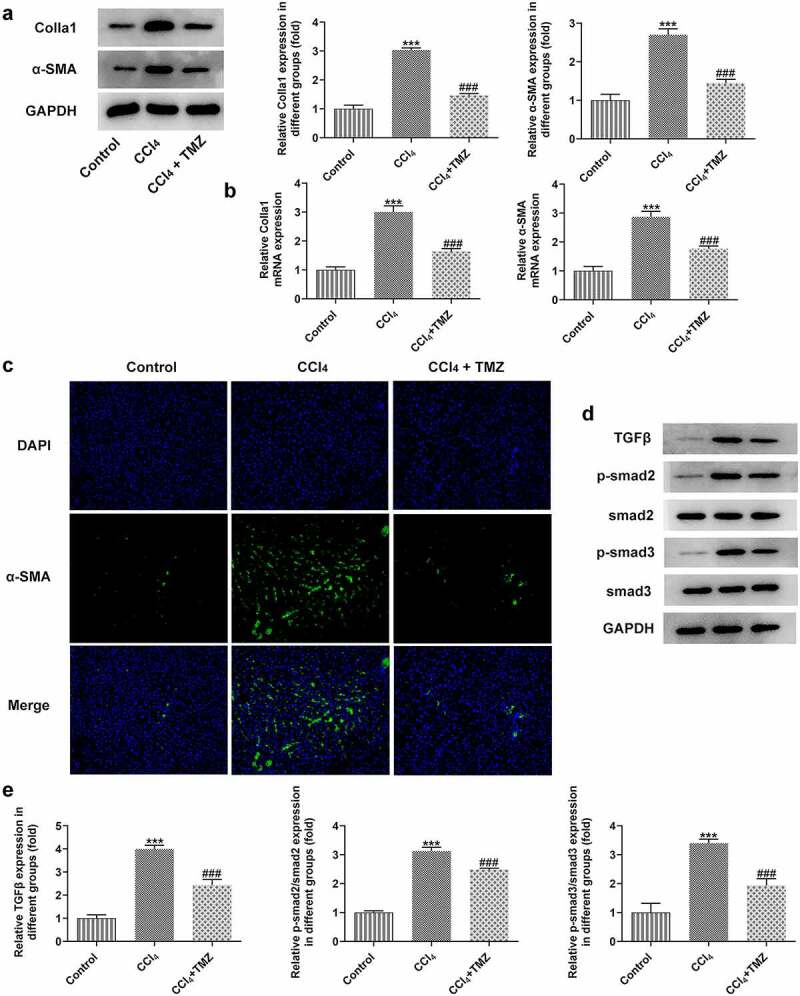
TMZ lessens the expression of extracellular matrix proteins and TGF-β/Smad signaling in CCl_4_-induced mouse tissues. (a) Western blotting and (b) RT-qPCR estimated colla1 and α-SMA expression. (c) α-SMA expression was tested by immunofluorescence. Expression levels (d) and semi-quantitative analysis (e) of TGF-β, smad2 and smad3. ***P < 0.001 vs. control; ^###^*P* < 0.001 vs. CCl_4._

In addition, the protein and mRNA expression levels of colla1 and α-SMA in tissues detected by western blotting and RT-qPCR were significantly increased after CCl_4_ induction, but this influence was markedly suppressed after treatment with TMZ (Figure 2a and b). Similarly, analogous results were observed upon immunofluorescence staining of tissues for α-SMA (Figure 2c). Meanwhile, to further investigate the mechanism of TMZ, the study analyzed the TGFβ/Smad signaling-related proteins by using western blotting. The results showed that TGFβ displayed elevated expression in liver tissues after CCl_4_ induction relative to the control group. In addition, TGFβ expression was inhibited after TMZ treatment compared with the CCl_4_ group. And the expression levels of smad2 and smad3 were consistent with the trend of TGFβ (Figure 2d–e).

### TMZ inhibits cell proliferation, promotes cell cycle arrest and downregulates extracellular matrix protein expressions in TGF-β-induced hepatic stellate cells

For the purpose of the investigation into the role of TMZ in hepatic stellate cells, functional experiments *in vitro* were performed using TGF-β-induced hepatic stellate cell lines from mice and human (JS-1 and LX-2 cells) for further analysis.

**Figure 3.**
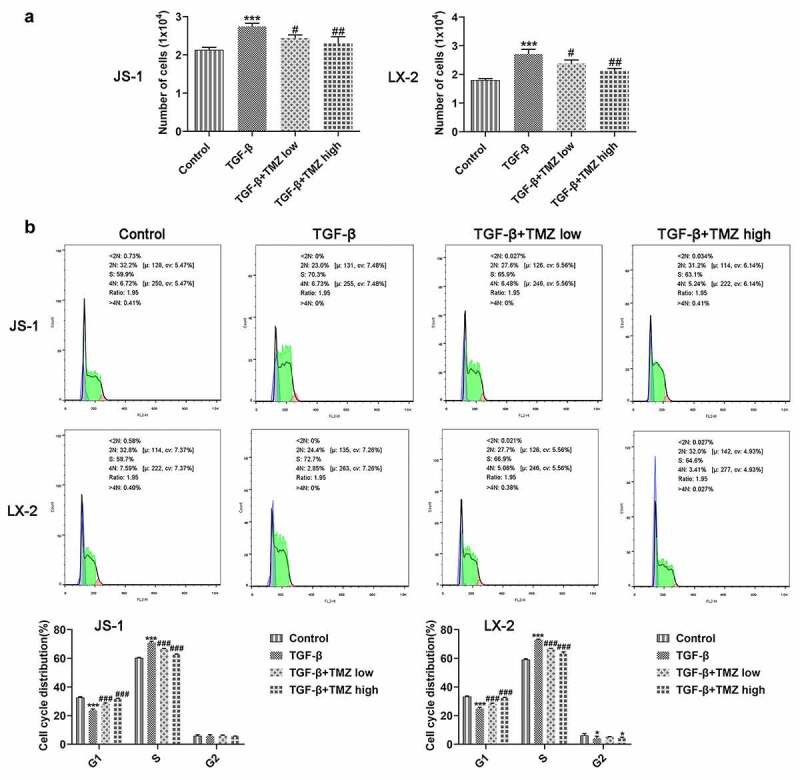
TMZ impedes proliferation and aggravates cell cycle arrest in TGF-β-triggered hepatic stellate cells. (a) Cell counting method to detect proliferation of JS-1 and LX-2 cells. (b) Flow cytometry detection of JS-1 and LX-2 cell cycles. *P < 0.05 and ***P < 0.001 vs. control; ^#^*P* < 0.05, ^##^*P* < 0.01 and ^###^*P* < 0.001 vs. TGF-β.

**Figure 4.**
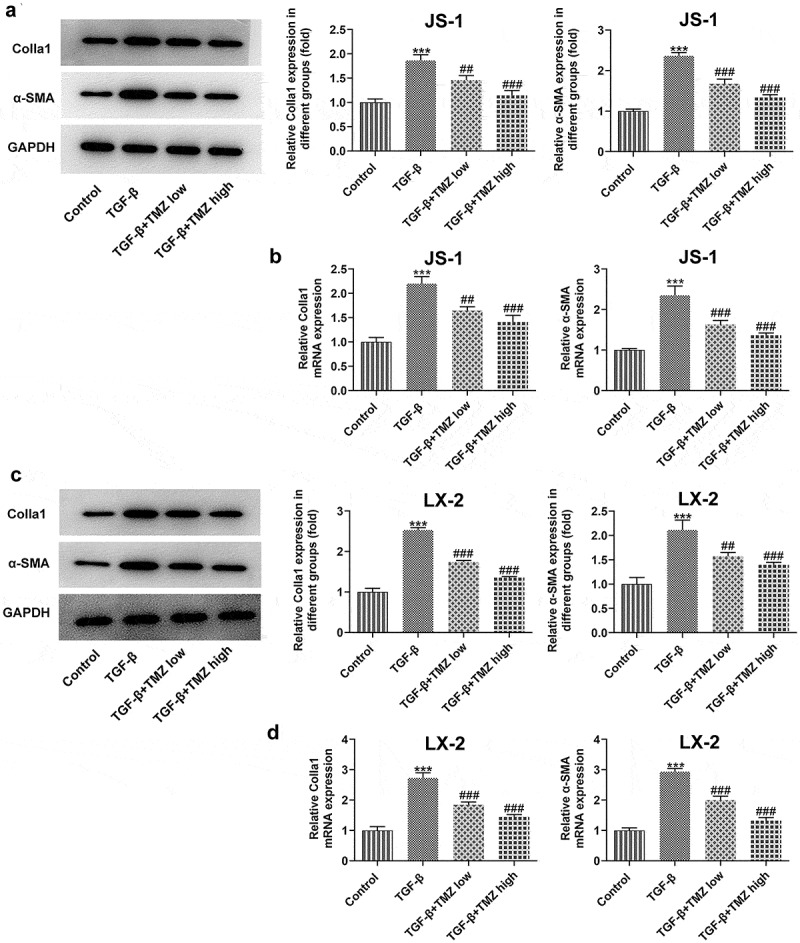
TMZ inhibits the expression of extracellular matrix proteins in TGF-β-induced hepatic stellate cells. The protein (a) and mRNA (b) expression levels of colla1 and α-SMA in TGF-β-induced JS-1 cells. α-SMA expression at protein level (c) and mRNA level (d) in TGF-β-induced LX-2 cells. ***P < 0.001 vs. control; ##P < 0.01 and ###P < 0.001 vs. TGF-β.

As exhibited in Figure 3a, the proliferation of JS-1 and LX-2 cells was boosted dramatically after TGF-β induction; however, TGF-β-induced cell proliferation was significantly inhibited in TGF-β+ TMZ group compared with TGF-β group. The cell cycle analysis revealed a significant increase in S phase cells after TGF-β induction, but the cell cycle converged to control group after TMZ addition (Figure 3b). Additionally, protein and mRNA expression levels of colla1 and α-SMA in TGF-β inducted JS-1 and LX-2 cells were detected by western blotting and RT-qPCR, respectively (Figure 4). The results illustrated that in JS-1 cell experiments, TGF-β induction caused a clear enhancement in protein and mRNA expression levels of colla1 and α-SMA as compared to control group. However, after TMZ treatment, the high expression of colla1 and α-SMA caused by TGF-β was dose-dependently inhibited by TMZ (Figure 4a and b). Similarly, the trends of TGF-β and TMZ effects on colla1 and α-SMA in LX-2 cell experiments were consistent with those in JS-1 cells (Figure 4c and d).

### TMZ inhibits cell migration and TGFβ/Smad signaling in liver fibrosis cell model

Cell migration ability was examined by Transwell assay, followed by further study of TGFβ/Smad signaling pathway protein expression levels in hepatic stellate cells.

**Figure 5.**
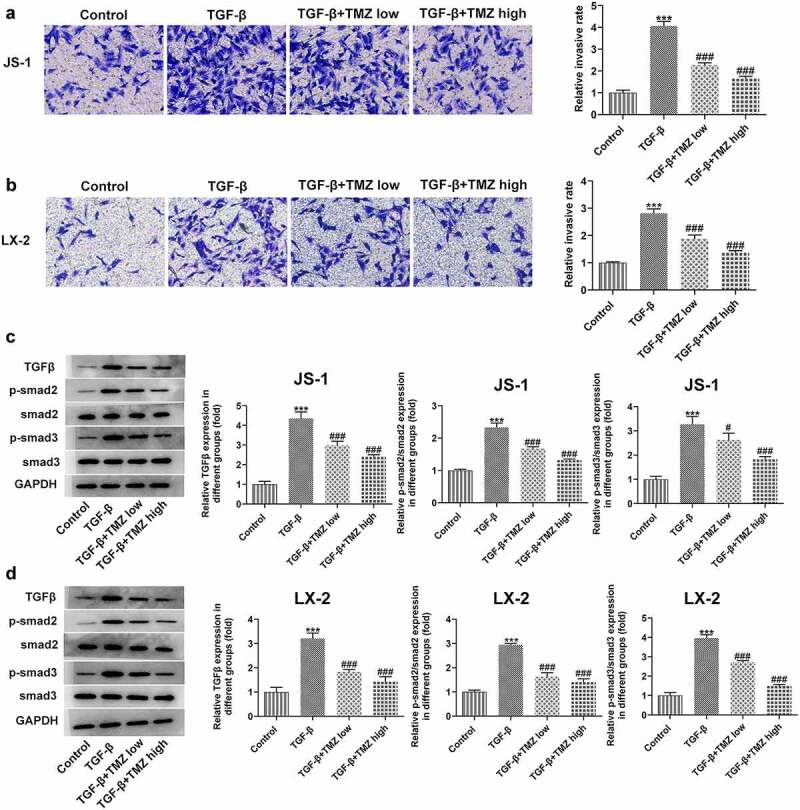
TMZ inhibits cell migration and inactivates TGF-β/Smad signaling in TGF-β-induced hepatic stellate cells. Results of cell migration analysis in TGF-β-induced JS-1 (a) and LX-2 cells (b). TGF-β, smad2 and smad3 expression in TGF-β-induced JS-1 (c) and LX-2 cells (d). ***P < 0.001 vs. control; ^#^*P* < 0.05 and ^###^*P* < 0.001 vs. TGF-β.

The experimental results from Transwell assay demonstrated that after TGF-β treatment, the migration abilities of JS-1 and LX-2 cells were both observably strengthened; nevertheless, the cell migratory behavior was inhibited after TMZ treatment. Meanwhile, to investigate the mechanism of TMZ action, the expression levels of TGFβ/Smad signaling pathway proteins were investigated (Figure 5a and b), which were consistent with the findings of in vivo experiments above. After TGF-β induction, smad2 and smad3 expression levels were upregulated; but after TMZ treatment, the upregulation level of smad2 and smad3 expression was suppressed (Figure 5c and d). Thus, it indicated that TMZ could interrupt the TGFβ/Smad signaling pathway.

## Discussion

The piperazine derivative TMZ has been used in the treatment of coronary heart disease, in addition to liver and kidney-related disorders. For instance, TMZ attenuated fibrosis, apoptosis and exacerbated autophagy in diabetic cardiomyopathy [23]; TMZ had protective effects against HgCl_2_-induced kidney injury by reducing oxidative stress-mediated [24]; TMZ improved adipogenesis and steatosis in nonalcoholic fatty liver via the AMPK-ChREBP pathway [25]. Furthermore, chronic liver disease often occurs as a result of chronic liver injury and inflammatory response [2]. And liver disease progression is greatly influenced by liver fibrosis [5]. In the present study, the effect of TMZ on liver fibrosis was investigated for the first time through a series of experiments using *in vivo* and *in vitro* liver fibrosis models.

The current research established a mice liver fibrosis model via CCl_4_ induction. The results showed that liver fibrosis features appeared in masson stained sections of liver tissue after CCl_4_ induction compared with control group. Additionally, the expression levels of liver enzyme ALT and AST in mouse serum were distinctly higher than those in the control group. These results illustrated that we successfully constructed an animal model of liver fibrosis. Besides, the expression levels of colla1 and α-SMA protein in liver tissues were markedly elevated, and the trends of these indicators were consistent with those reported by previous investigators [26,27]. More importantly, TGFβ and smad protein expression levels were prominently high in the mice liver fibrosis model, suggesting the activation of TGFβ/Smad signaling in the process of liver fibrosis. Encouragingly, TMZ significantly suppressed liver fibrosis in mice model, including downregulation of liver enzyme (ALT and AST) expression levels and extracellular matrix protein (colla1 and α-SMA) expression levels. Furthermore, TMZ was found to significantly inhibit TGFβ/Smad signaling expression. As such, TMZ showed a clear inhibitory effect on liver fibrosis and blocked the expression of TGFβ/Smad signaling *in vivo*.

This study was also explored *in vitro* liver fibrosis model established by TGF-β-induced hepatic stellate cells. The cell proliferation, cycling and migration behavior of hepatic fibroblasts were investigated by cell counting, flow cytometry and transwell, respectively. Studies revealed an increase in cell proliferation and migration of TGF-β-induced hepatic stellate cells. This is consistent with the previously reported trend [28].

Specifically, the results showed that both 10 μM and 40 μM TMZ significantly inhibited TGF-β-induced proliferation and migration of human and murine-derived hepatic stellate cells, and promoted cell cycle arrest. Notably, the influences of TMZ on extracellular matrix proteins and TGFβ/Smad were consistent with the *in vivo* results, that is, the protein expression levels were both inhibited by TMZ.

More importantly, it has been reported that TGFβ/Smad signaling pathway has been confirmed to be an important factor in the development of liver fibrosis [29]. Combined with the above findings, TMZ was able to effectively block TGFβ/Smad signaling *in vitro* and *in vivo*. Therefore, TMZ may be valued as an effective compound in liver fibrosis therapy.

## Conclusion

In the present study, we investigated the effects of TMZ *in vitro* and *in vivo* by constructing liver fibrosis models with CCl_4_-induced mice and TGF-β-induced hepatic stellate cells, respectively. It was revealed that TMZ suppressed liver fibrosis, hepatic stellate cell proliferation, and blocked TGFβ/Smad signaling *in vitro and in vivo*. This might provide a novel therapeutic direction for the treatment of liver fibrosis.

## Highlights

1. TMZ inhibits the TGF-β/Smad signaling in CCl_4_-induced mice liver fibrosis.
2. TMZ promotes cell cycle arrest in TGF-β-induced hepatic stellate cells.
3. TMZ inhibits TGF-β-induced extracellular matrix protein expression levels.
4. TMZ inactivates TGF-β/Smad signaling in the cell model of liver fibrosis.
